# Application of different measures of skeletal maturity in initiating weaning from a brace for scoliosis: two case reports

**DOI:** 10.1186/1752-1947-3-6444

**Published:** 2009-04-01

**Authors:** LouAnn Rivett, Alan Rothberg, Aimee Stewart, Rowan Berkowitz

**Affiliations:** 1 Physiotherapy Department, School of Therapeutic Sciences, Faculty of Health Sciences, University of the Witwatersrand, PO Box 2398, Randburg, 2125, South Africa; 2 Sandton, Sunninghill and Morningside Clinics, Johannesburg, South Africa

## Abstract

**Introduction:**

Various measures of skeletal maturity are used to initiate weaning from a brace in patients suffering from idiopathic scoliosis, resulting in different outcomes. We present two cases with double major curves, treated with the Rigo System Cheneau brace, and weaned using different criteria.

**Case presentation:**

Case 1 was a South African, Caucasian girl who was initially treated with a brace at 14.75 years and who began weaning at 16.25 years on the basis of the Greulich and Pyle Index. She was out of her brace in 6 months, at least 11 months before reaching skeletal maturity as shown by the Risser Sign. Case 2 was a South African, Caucasian girl, initially treated with a brace at 14.25 years and who began the weaning process at 17.67 years on the basis of skeletal maturity according to the Risser Sign and static height for a period of 6 months. She was out of the brace 12 months later. In Case 1, the thoracic Cobb angle progressed during weaning and scoliometer readings deteriorated. The iliac apophysis fused 11 months after the wrist. In Case 2, the therapeutic gains made during the period of bracing were maintained during weaning, that is the improvement in the lumbar Cobb angle was maintained until the brace was removed, and scoliometer readings improved. The iliac apophysis fused 8.5 months after the wrist.

**Conclusions:**

In patients with idiopathic scoliosis, it would seem to be more appropriate to base the timing of weaning on the Risser Sign and static height measurements rather than on traditional methods such as the Greulich and Pyle Index.

## Introduction

Adolescent Idiopathic Scoliosis (AIS) is a worldwide condition affecting 2% to 3% of adolescents [1]. Lonstein and Carlson describe it as lateral curvature of the spine in an otherwise healthy child, for which the cause is unknown [2]. The frequency of AIS is similar in boys and girls, however, progression is more common and also more severe in girls. Scoliosis itself is defined as a three-dimensional deformity where the spine deviates from the normal sagittal and coronal positions in upright posture, with the potential to develop into a fixed and unbalanced posture [3]. Kotwicki *et al.* go further to say that scoliosis is a three-dimensional deformity of the spine and of the trunk [4].

In South Africa, management of adolescents with AIS is usually directed by an orthopaedic surgeon, with referral to other disciplines for investigation and supportive care. The physiotherapist plays a leading role in terms of the latter, but from the perspective of one (LR) who has undergone specialised training and has treated some 170 affected adolescents over a period of 20 years, there remain some vexing questions. In the vast majority of cases, it will be the orthopaedic surgeon who will recommend if or when bracing is required, and will also determine the need for, and timing of, corrective surgery. While there are various bracing options, the system that is consistently used within the abovementioned group of 170 patients is the Rigo System Cheneau (RSC) brace. A full understanding of the technical rationale for using this brace requires an understanding of the three-dimensional nature of AIS deformities, and the clinical measurements involved (Table 1).

**Table 1:** Conventional clinical measurements used in the management of AIS patients

Measurement	Description	Comment
Cobb angle	The degree of tilt between two vertebrae (caudal and cranial end vertebrae) that are the most tilted on radiological examination [5]	This expresses the magnitude of lateral deviation of the curve
Angle of rotation of apical vertebra	On X-ray, this is the most translated and rotated vertebra in the transverse plane. Measurement in these cases was with the Perdriolle torsiometer	Vertebral rotation tends to increase with increasing Cobb angle
Scoliometry	A scoliometer (in these cases, the Bunnell scoliometer) measures the angle of trunk rotation, not vertebral rotation. Readings are taken in the sitting, forward bending position, so it is recommended as it provides stable posture and eliminates limb discrepancy [4]	Scoliometer readings on their own may be misleading and are not related to radiological data (Cobb angle and apical rotation). Both modalities should be considered in planning and evaluation of scoliosis treatment [6]
Kyphosis and lordosis angles	These are measured on sagittal view X-rays using the Cobb method (T4-T12 for kyphosis; L1-L5 for lordosis)	These measurements are taken as scoliosis may be associated with loss of normal sagittal curves [7]
Peak Expiratory Flow (ml/s)	Subjects inhale maximally and then exhale forcibly and as quickly as possible into a spirometer (in this case, the Mini-Wright Peak Flow meter). Subjects blow into the meter three times, with 30s breaks between attempts. The best of three results is taken	Scoliosis leads to restrictive lung disease secondary to reduced chest wall compliance. Chest wall compliance and vital capacity are inversely correlated with Cobb angles >10 degrees. As Cobb angle and apical rotation increase, there is a decrease in peak expiratory flow, total lung capacity, vital capacity, and functional residual capacity [8]. Curves >40-50 degrees may cause cor pulmonale

The RSC brace is used particularly to prevent progression of Cobb angles >20 degrees and to reduce the extent of deformity, with the ultimate goal of minimising the number of patients requiring corrective surgery [9] (generally regarded as being necessary with Cobb angles > 50 degrees in skeletally-mature patients and >40 degrees in the immature [5,9]). Progression is a difference of greater than 5 degrees between two X-rays, and is used to document that a curve has deteriorated or improved [7]. Furthermore, in contrast to other braces, the RSC brace addresses the rotational component of scoliosis, and it not only works through compression, but also exploits the lung and breathing mechanics. The RSC brace corrects frontal and sagittal alignment in a three-dimensional way, with correction achieved through distortional forces. Breathing mechanics produce the necessary internally active forces, pushing out the sunken areas of the trunk as well as the sunken spine. The brace addresses, and is used to correct, the 15 curve patterns that make up the Rigo classification of scoliosis curves [10].

Despite the scientific rationale for bracing, there is still controversy as to the effectiveness of the procedure. Reliable estimates of the effectiveness of bracing have been problematic, largely because of variation in brace type and lack of standardisation in application [11]. In patients referred for bracing, a phased weaning process commences at the point at which the orthopaedic surgeon decides that skeletal maturation is complete. However, there is also debate and variation around this latter aspect of AIS management, with proponents of comprehensive assessment of maturity concerned that premature weaning may negate any beneficial effects of bracing.

The diagnosis of skeletal maturity is essentially based on radiological investigations. Radiologists in South Africa appear to rely mostly on the index devised by Greulich and Pyle for bone age assessment. Many orthopaedic surgeons wean their AIS patients on the basis of this index, which uses 'atlas matching' of an X-ray of the left wrist to assess bone age and skeletal maturity [12]. An alternative method of assessing skeletal maturity, using X-rays of the left wrist and hand, is that of Tanner and Whitehouse [13]. This method uses a point-scoring system instead of matching to an atlas. Several authors have noted the poor correlation between the two methods of bone age assessment, concluding that the Greulich and Pyle method is the less precise of the two [13]. While perhaps more cumbersome, the Tanner and Whitehouse method is more reproducible, mainly because it takes into account the fact that the bones of the hand and wrist do not necessarily mature at the same rate, and instead of having to make a judgement call as to the best 'atlas match,' Tanner and Whitehouse separated hand from wrist, assigning scores and skeletal maturity to individual bones. Furthermore, it has been reported that the digits are more reliable indicators of maturity than the carpus.

Having noted that the bones of the hand and wrist may differ in terms of skeletal maturity, the inevitable question is how well the hand and wrist will predict axial maturity. In this regard, a more appropriate system for assessing skeletal maturity in AIS appears to be assignment of a grade using the Risser Sign. This five-point grading system has been shown to be valuable in the determination of skeletal maturity and prediction of spinal curve progression [14]. While not used extensively in South Africa by orthopaedic surgeons or radiologists in determining when to initiate weaning from a brace, it has nevertheless been used by the first author (LR) as an adjunct to conventional measurements. The Risser Sign is defined by the amount of calcification in the iliac apophysis and tracks progressive anterolateral to posteromedial ossification. Risser 1 signifies ≤25% ossification, whereas at Risser 5, the iliac apophysis has fused to the iliac crest after 100% ossification. Several studies have evaluated the reliability of the Risser Sign, with most supporting the methodology [13,14]. The Risser Sign is considered a less reliable indicator in boys, with ossification starting relatively early with respect to further growth potential.

Adding to the problem of accurate staging of skeletal maturation is apophyseal image variation according to the radiological view. The appearance of the iliac apophysis on posterior-anterior X-rays cannot be used as a reliable indicator of skeletal maturity because the full length of the iliac apophysis cannot be adequately visualised. When compared to the more accurate anterior-posterior (A-P) views, there is a distortion of the iliac apophysis, with the medial and lateral aspects superimposed over the ilium. The authors (LR and RB) use a coned view, A-P including only the iliac crest to avoid irradiating the gonads.

The timing of weaning from a brace is important to physiotherapists treating adolescents with scoliosis, particularly since several have reported back to the referring orthopaedic surgeons that early weaning has been associated with reversal of gains achieved during bracing. Two cases are presented to illustrate this point.

## Case presentation

Both cases were initially treated with bracing on the instructions of the consulting orthopaedic surgeon, but were only referred to the specialist physiotherapist (LR) some time later. For reasons given below, bracing was continued. However, the point to be made in presenting these cases relates more to the different weaning criteria that were applied. The same assessor was responsible for all measurements in both cases (RB). There is evidence to show that intra-observer variation for a single X-ray tends to be <2 degrees, whereas interobserver variation can range from 2 to 10 degrees [7]. X-rays and measurements were taken at the same time of day. Both girls wore the brace for ±20 hours a day (as recorded in a diary that is a standard requirement in the physiotherapy practice involved in this report). Standard treatment also involved a specific set of exercises that has been developed by this practice and is the subject of a separate research report. An information leaflet on scoliosis and its benign nature was given to patients and parents. Ethical clearance was obtained from the Committee for Research on Human Subjects at the University of the Witwatersrand (Reference M060702).

### Case 1

At 14.75 years of age, this girl was first diagnosed with scoliosis. According to the Rigo classification, she suffered from a double major thoracic and lumbar curve [10]. Thoracic Cobb angle was 28 degrees (T3-T11) and lumbar Cobb angle was 15 degrees (T12-L4). She was Risser 2 and menarche had been at 13 years. She was referred for bracing by the orthopaedic surgeon despite not qualifying for a brace according to best practice guidelines [1]. Her progression risk was 50% and a scoliosis intensive rehabilitation program was not offered to her. A Boston brace was worn for 11 months, after which it was removed because of pain and discomfort. The pain continued and she was unhappy with her cosmetic outcome. Following consultation with the orthopaedic surgeon, the young girl was referred to this practice and treated by LR. An RSC brace was applied together with physiotherapy. Indication for the brace at this stage (15.67 years) was presentation of an adolescent with scoliosis and chronic pain (SOSORT guideline category VI [1]). Sexual and skeletal maturity measurements were at Tanner 4 and Risser 4, respectively. Measurements taken immediately before RSC bracing are shown in Table 2.

**Table 2:** Clinical measurements in Case 1 at onset of RSC bracing and subsequently

	Initial measurements	Weaning after 7 months	At Risser 5
Cobb angle (degrees)			
- Thoracic	19 (T3-T11)	20	26
- Lumbar	21 (T12-L4)	17	18
Rotation of apical vertebra (degrees)			
- Thoracic	10(T8)	10	10
- Lumbar	15 (L1)	15	15
Scoliometer (degrees)			
- Thoracic	7	5	7
- Lumbar	5	3	4
Peak flow (ml/s)	390	470	480
Height (cm)	159.3	158	159
Kyphosis (degrees)		24	31
Lordosis (degrees)		50	51

After 7 months of wearing the brace, the treating orthopaedic surgeon gave instructions to initiate weaning on the basis of the Greulich and Pyle Index. While the wrist was totally fused, iliac examination showed a Risser Sign of 4+. At the start of weaning, the lumbar Cobb angle had improved by 4 degrees, scoliometer readings had improved slightly, and there was a 20.5% improvement in peak flow. However, during the weaning process, the Cobb angle deteriorated by 6 degrees and scoliometer readings reverted to baseline levels. The patient was completely out of the brace after a further 6 months, at which point she had not yet reached Risser 5. In other words, there was at least an 11-month discrepancy in maturation between the Greulich and Pyle and Risser measures. While one might have expected some linear growth in a woman who had not yet reached skeletal maturity, the absence of any change in height can be attributed to the progressive spinal deformity. The patient was pain free at the end of brace weaning.

### Case 2

At 14.25 years of age, this girl was diagnosed with scoliosis (a double major thoracic and lumbar curve). Thoracic Cobb angle was 24 degrees (T4-T10) and lumbar Cobb angle was 30 degrees. She was Risser 0 and menarche had been at 13 years. These parameters placed her at 90% risk of progression, according to category IIe of the SOSORT guidelines [1] and she was referred for treatment with a Milwaukee brace. At 16 years, she had been weaned from the Millwaukee brace, but thoracic Cobb angle then progressed from 11 to 15 degrees and lumbar Cobb angle progressed from 18 to 24 degrees. The parents and girl were very unhappy with this result and her cosmetic outcome, and were concerned about further progression. They consulted another surgeon who referred her to this practice. She was then put into an RSC brace with physiotherapy (at 16.8 years). Sexual and skeletal maturity measurements placed her at Tanner 4 and Risser 4+, respectively. Measurements taken before going into the RSC brace are shown in Table 3.

**Table 3:** Clinical measurements in Case 2 at onset of RSC bracing and subsequently

	Initial measurements	Weaning after 10 months and at Risser 5	Final measurements after weaning for 12 months
Cobb angle (degrees)			
- Thoracic	15 (T4-T10)	13	15
- Lumbar	24 (T11-L4)	17	17
Rotation of apical vertebra (degrees)			
- Thoracic	10 (T8)	10	10
- Lumbar	15 (L2)	5	10
Scoliometer (degrees)			
- Thoracic	13	6	6
- Lumbar	4	5	5
Peak flow (ml/s)	400	440	440
Height (cm)	161.2	161.6	161.7
Kyphosis (degrees)	33	33	33
Lordosis (degrees)	35	45	43

A wrist X-ray after 6 weeks of wearing the brace showed that the radius had fused, however, the orthopaedic surgeon permitted full-time bracing to continue (20 hours per day) for 10 months. Initiation of weaning (based on Risser 5 and static height for 6 months) was started after 10 months of bracing and exercise, and continued over the subsequent 12 months. Table 3 shows that, in this patient, the progress made during full-time bracing was maintained during the weaning process. Final measurements showed improvements from baseline in lumbar Cobb angle and rotation, scoliometer readings, and peak flow. However, there was some worsening of the lordosis when compared against baseline. Finally, the patient was happy with her result and cosmetic outcome.

## Discussion

Results of conservative management programmes (typically involving bracing and exercises) include improved postural balance, a reduction in vertebral rotation [8], decreased pain, cosmetic and often actual improvement in the extent of deformity, increased chest expansion and vital capacity, improvement in psychological distress, and a reduced rate of curve progression [9]. Skeletal maturity represents a point beyond which one is unlikely to continue to modify the pathological process, but as pointed out in the introductory section, the correct timing of the weaning process is a subject of debate and controversy.

In Europe, there is no consensus about weaning. For example, Rigo in Spain weans at around Risser 4+ and, provided that patients are compliant, continues with bracing for 16 hours per day for 6 to 12 months. Hoppenfeld *et al.* found that the mean linear growth rate of girls after Risser 4 was 1.75cm and of boys was 2.46cm [15]. They found no growth after Risser 5, or after closure of the rib epiphyses or proximal humeral growth plates, and therefore concluded that other growth centres should be evaluated in conjunction with serial height measurements in the determination of skeletal maturity. The upshot of the above is that the data indicate that bracing may be required for longer than previously thought, and as communicated by Rigo (personal communication), this is not easily accepted by late adolescents.

According to the management diaries kept by the two patients in this report, compliance was good during both 'full-time' bracing (targeted at 20 hours per day) and during the weaning processes. Compliance on its own is important in preventing progression of scoliosis, while the combination of compliance and initial correction has been shown to improve Cobb angle by an average of 7 degrees [16]. However, the variable under discussion here is the timing of initiation of the weaning process. In the first case, the patient was weaned according to the Greulich and Pyle Index and, as shown in Table 2, clinical gains made during the first phase appeared to be lost during the weaning process. There was at least an 11-month gap between fusion of the wrist and fusion of the iliac apophysis, the patient's thoracic Cobb angle progressed during weaning, and she was out the brace before reaching Risser 5. In the second case, weaning was commenced at Risser 5 and static height for 6 months, and therapeutic gains were maintained after complete weaning. Lumbar Cobb angle improved by 7 degrees. and scoliometer readings, lumbar apical rotation and peak flow all improved (Table 3). The difference between wrist maturation and iliac maturation was also present in this patient, with the wrist fused 8.5 months before reaching Risser 5.

## Conclusions

Reliable methods are needed to assess skeletal maturity because premature weaning from a brace can reverse clinical gains that have been achieved. It would appear that no single method of determining skeletal maturity is completely reliable in deciding when to initiate weaning. The Tanner and Whitehouse method appears to be superior to that of Greulich and Pyle, but iliac ossification and apophyseal maturity as proposed by Risser would seem to be more appropriate since they involve sites that are closer to the area of interest. Even so, there are data to suggest that additional information should be sought from other growth sites, and that serial height measurements should be included. Weaning from a brace should not be a hurried process, but it is acknowledged that compliance can be problematic in AIS patients. On the other hand, our experience is that in a supportive environment, it is indeed possible to facilitate compliance well into the late teens.

## Consent

Written informed consent was obtained from the patients and their parents for publication of this case report and any accompanying images. A copy of the written consent is available for review by the Editor-in-Chief of this journal.

## Competing interests

The authors declare that they have no competing interests.

## Authors' contributions

LR designed the study, applied the exercise routine, acquired, analysed and interpreted the data, drafted the manuscript, and gave final approval of the version to be published. AR interpreted the data, revised the manuscript, and gave final approval of the version to be published. AS assisted with study design and drafting of the manuscript, and gave final approval of the version to be published. RB assisted with study design, collection and interpretation of data, and gave final approval of the version to be published.
